# Editorial: Fire effects on native plant species diversity: patterns and mechanisms

**DOI:** 10.3389/fpls.2026.1910907

**Published:** 2026-08-13

**Authors:** Sofía Gonzalez, Luciana Ghermandi, Oscar Cruz de la Fuente, Sabine Kasel, Setsuko Komatsu, Ya Fei Shi

**Affiliations:** 1 Institute for Research in Biodiversity and the Environment, National University of Comahue, Bariloche, Argentina; 2 University of Santiago de Compostela, Santiago de Compostela, Spain; 3 School of Agriculture, Food and Ecosystem Sciences, The University of Melbourne, Burnley, Australia; 4 Fukui University of Technology, Fukui, Japan; 5 College of Grassland Science, Gansu Agricultural University, Lanzhou, China

**Keywords:** ecosystem resilience, fire-cues, post-fire recovery, recruitment, resprouting

Wildfire plays a key role in maintaining and enhancing biodiversity in ecosystems worldwide (Keeley and Pausas, 2022). Post-fire vegetation patterns typically feature the resprouting of fire-tolerant species and the widespread recruitment of fire-ephemeral plants (Ghermandi et al., 2004). The release of resources such as space and light following fire promote vegetation resprouting and seed germination (He et al., 2019). Moreover, fire-related cues, such as heat and smoke can stimulate germination from the soil seed bank, thereby promoting plant diversity (Moreira et al., 2010; Keeley and Pausas, 2018). Understanding the mechanisms behind these post-fire diversity patterns is essential for improving conservation, restoration, and sustainable land management.

This Research Topic comprises studies that identify post-fire patterns that enhance the native plant diversity and the mechanisms that explain these patterns. It addresses how fire affects plant diversity across multiple ecological scales, from vegetation recovery at community level to the ecological mechanisms that determine species persistence and regeneration. The collection includes six articles by 30 authors, providing a comprehensive overview of current advances in this field. Several contributions were conducted in Mediterranean type-climate ecosystems, including northwestern Patagonia in Argentina, central Chile, southern California, and the sub-Mediterranean region of southwestern Slovenia. These ecosystems are strongly shaped by fire and are experiencing increasing wildfire frequency and intensity under ongoing climate change, highlighting the importance of understanding how native plant communities and species respond to changing fire regimes (Keeley et al., 2012).

Two studies focused on vegetation response to fire. At the community level, the study by Carni et al. documented post-fire succession in sub-mediterranean forest vegetation on the Kras Plateau in southwestern Slovenia over a three-year period. Because fire has historically been uncommon in this region, the vegetation is considered poorly adapted to this disturbance. Despite this, the authors found that the community showed high resilience to wildfire, characterized by early dominance of ephemeral and ruderal species, which rapidly declined as the abundance of resprouting species increased. This study highlighted that enhanced autogenic succession, combined with favorable post-fire moisture conditions, can facilitate rapid ecosystem recovery, potentially reducing the risk of long-term degradation or the establishment of persistent post-fire shrublands.

For the endangered *Nothofagus alessandri* forests in central Chile Leal-Medina et al. assessed post-fire responses following the 2017 “Las Máquinas” megafire. They found rapid vegetation recovery through resprouting, although increasing fire severity promoted the invasion of *Pinus radiata*, highlighting how severe fires can accelerate forest degradation and threaten the persistence of native ecosystems.

At the landscape-scale, Davis et al. evaluated the potential impacts of climate change and wildfire on the future distributions of *Pseudotsuga macrocarpa* and *Quercus chrysolepis* in southern California. Species distribution and wildfire probability models indicated significant habitat contractions for both species and increased wildfire probability throughout the 21^st^ century. Areas currently functioning as topoclimatic refugia may become increasingly exposed to fire, reducing their capacity to support long-term persistence. The projected decline of *Q. chrysolepis*, a species that currently limits fire spread and severity, may reduce the capacity of these landscapes to buffer *P. macrocarpa* from severe wildfires. The authors emphasize the need to prioritize fuel reduction and post-fire restoration efforts.

Three studies focused on the ecological mechanisms that influence post-fire germination, seedling recruitment, and establishment of native plant species. Gonzalez et al. investigated germination responses of two native ephemeral annuals *Boopis gracilis* and *Nicotiana linearis* to smoke water and karrikinolide treatments in northwestern Patagonian grasslands, Argentina. By testing different concentrations, they found that karrikinolide promotes seed germination, whereas smoke water inhibited it. These findings improve the understanding of the mechanisms underlying the massive post-fire germination of ephemeral species observed in these grasslands.

Although fire-related cues can stimulate germination, vegetation recovery ultimately depends on the successful establishment and growth of seedlings in post-fire environments. Chen et al. conducted one of the first experimental assessments of post-fire establishment in native Alpine conifers. They evaluated the effects of seed exposure to fire and sowing time under post-fire conditions on the establishment of *Pinus cembra*, *Picea abies*, and *Larix decidua* in Tyrol, Austria. Their results showed marked interspecific differences in post-fire establishment, with *P. cembra* successfully established when seeds were sown immediately after fire. Finally, in south-central Chile, Diaz-Mons et al. planted seedlings of the native conifer *Araucaria araucana* in forests affected by different levels of fire severity. The authors assessed the role of biological legacies (e.g., fallen logs, standing dead trees) and cattle on the seedling survival and growth. Biological legacies and cattle exclusion enhanced seedling height growth, particularly in severely burned forests, highlighting the potential post-fire management practices in supporting forest recovery.

The articles compiled in this Research Topic advance the understanding of post-fire vegetation patterns and the mechanisms underlying these responses. Together, they provide a broader perspective of how fire shapes biodiversity and ecosystem resilience. At the community level, rapid recovery through recruitment and resprouting can support the persistence of native vegetation, whereas increasing fire severity and biological invasions may threaten biodiversity and accelerate ecosystem degradation. At the species level, responses to fire-related cues, post-fire environmental conditions, and biological legacies affect seed germination, seedling establishment, and survival, ultimately shaping post-fire plant communities. As climate change increases fire frequency and intensity, ecosystems are experiencing unprecedented impacts with important consequences for biodiversity. Future studies should evaluate how changing fire regimes interact with climate change, biological invasions, and land-use to influence native plant diversity over longer time scales. Such investigations will improve predictions of post-fire vegetation trajectories and help guide conservation and restoration strategies in fire-prone ecosystems.
